# Associations between the prenatal exercise environment, exercise experience, and adherence: a cross-sectional study of affective and cognitive pathways

**DOI:** 10.3389/fpubh.2026.1933490

**Published:** 2026-09-17

**Authors:** Mingmao Li, Hongli Yu, Chen Sun, Zhaoxiang Zhang, Zbigniew Ossowski

**Affiliations:** 1 Faculty of Physical Culture, Gdansk University of Physical Education and Sport, Gdansk, Poland; 2 College of Physical Education, Sichuan University of Science & Engineering, Zigong, Sichuan, China; 3 College of Physical Education and Health Science, Yibin University, Yibin, Sichuan, China

**Keywords:** exercise environment, physical activity, prenatal exercise, psychological distress, repetitive negative thinking

## Abstract

**Introduction:**

Prenatal exercise has established health benefits, yet adherence remains low, and the role of the exercise environment in shaping psychological experience during pregnancy is unclear. This study aimed to examine the associations of the prenatal exercise environment with exercise experience and psychological distress, with particular attention to the proposed affective and cognitive pathways within the Prenatal Exercise Environment Dual-pathway framework.

**Methods:**

In this cross-sectional study, 361 pregnant women were recruited through multicenter convenience sampling from medical centers in five regions of China. The researcher-developed Prenatal Exercise Environment and Experience Questionnaire underwent preliminary psychometric evaluation in the current sample, followed by structural equation modeling with 5,000 bootstrap resamples and machine-learning analyses.

**Results:**

The prenatal exercise environment was positively associated with positive affective state (β = 0.328, *p* < 0.001) and exercise enjoyment (β = 0.270, *p* < 0.001) and negatively associated with repetitive negative thinking (β = −0.297, *p* < 0.001). Positive affective state was positively associated with exercise enjoyment (β = 0.143, *p* = 0.007), whereas repetitive negative thinking was negatively associated with exercise enjoyment (β = −0.216, *p* < 0.001). Exercise enjoyment was inversely associated with psychological distress (β = −0.253, *p* < 0.001). The model explained 21.8% of the variance in exercise enjoyment but only 6.4% in psychological distress, indicating limited explanatory power for the latter. Exploratory machine learning identified the sensory and physical environment as the strongest predictor of weekly Thermic Effect of Activity.

**Conclusion:**

Prenatal exercise environments influence psychological outcomes through dual affective and cognitive pathways. Improving environmental quality and reducing negative cognition may enhance exercise engagement and mental well-being during pregnancy.

## 1 Introduction

Moderate prenatal exercise has been extensively demonstrated to confer significant physical and psychological benefits, such as reducing the risks of gestational diabetes and hypertension, controlling gestational weight gain, and alleviating anxiety and depressive symptoms (1, 2). Even though pregnant women are strongly encouraged to participate in at least 150 min of moderate-intensity aerobic exercise each week by the American College of Obstetricians and Gynecologists (ACOG) (3), only about 15–25% of women meet this target in practice (4). Poor adherence to prenatal exercise represents a major barrier to health promotion, driven by physical discomfort, fatigue, and more complex psychological and environmental factors (5). Previous research on physical activity during pregnancy has primarily focused on individual-level and interpersonal barriers and facilitators, including physical discomfort, fatigue, perceived safety, pregnancy-related concerns, and social support (5, 6). In addition, systematic evidence has identified self-efficacy, exercise intention, and mental health as important correlates of prenatal physical activity (7). Although these studies have substantially advanced understanding of why pregnant women engage in or withdraw from physical activity, environmental characteristics have generally been treated as isolated contextual facilitators or barriers rather than as a multidimensional construct. More recent pregnancy-specific research has begun to examine environmental exposures; for example, objectively assessed greenspace and walkability have been associated with physical activity during pregnancy (8). However, such studies have predominantly focused on neighborhood or built-environment characteristics and activity outcomes rather than pregnant women’s perceptions of sensory, spatial, privacy-related, and social characteristics within exercise settings.

Psychological pathways have also largely been examined separately from these environmental factors. For example, self-efficacy has been investigated as a mediator linking anxiety, social support, and other psychosocial factors with physical activity during pregnancy (9). These studies provide important evidence regarding individual motivational and psychological determinants, but they do not establish whether multidimensional perceptions of the exercise environment are simultaneously associated with exercise experience through distinct affective and cognitive processes. Thus, what remains unresolved is not only the absence of an integrated framework, but also how sensory, spatial, and social environmental perceptions relate to positive affect and repetitive negative thinking, and how these processes are associated with exercise enjoyment, psychological distress, and activity-related outcomes during pregnancy.

In environmental psychology and health behavior research, environmental characteristics are theorized to be associated with behavioral decisions by modulating affective responses, attentional focus, and cognitive appraisal (10). Empirical studies have shown that multisensory stimulus such as music, lighting, color, and spatial design—can substantially alter emotional responses and subjective experience in fitness, rehabilitation, or hospital settings (11). One study further demonstrated that environmental elements influence perception and evaluation through dual mechanisms of “emotional regulation” and “attentional-memory pathways” (12). However, these findings cannot be assumed to apply directly to prenatal exercise, because pregnancy involves distinctive physiological changes, safety concerns, and heightened sensitivity to bodily sensations. Pregnant women may also experience elevated anxiety and attentional bias related to bodily changes and perceived risks, making the exercise environment a potentially relevant contextual factor in exercise experience and adherence. Moreover, the environmental psychology and prenatal physical activity literatures have largely developed separately. Existing prenatal exercise studies have rarely examined multidimensional environmental perceptions together with affective and cognitive processes, exercise enjoyment, psychological distress, and activity-related outcomes within a single analytical framework.

Repetitive Negative Thinking (RNT) refers to a persistent and uncontrollable pattern of cognitive processing characterized by repetitive focus on negative events, worries, or bodily sensations (13). RNT is recognized as a core maintaining mechanism of emotional disorders that increases psychological burden and depletes attentional resources (14). During pregnancy, RNT has been associated with heightened anxiety, perceived stress, and diminished motivation for physical activity (15).

In prenatal exercise settings, environments perceived as uncomfortable, unpredictable, unsafe, lacking privacy, or insufficiently supportive may be associated with heightened vigilance toward bodily sensations and pregnancy-related concerns. Such cognitive vigilance may make disengagement from worry and repetitive negative thoughts more difficult, whereas more favorable environmental perceptions may be associated with fewer situational cues that sustain RNT. In contrast, a more positive affective state may be associated with greater exercise enjoyment, whereas RNT may be associated with less favorable exercise experiences (16). These contrasting processes provide a theoretical basis for examining positive affect and RNT as parallel affective and cognitive pathways.

Accordingly, the principal research gap is not simply a general lack of attention to the prenatal exercise environment, but the limited integration of multidimensional environmental perceptions with parallel affective and cognitive processes. To address this gap, the present study is guided by the proposed Prenatal Exercise Environment Dual-pathway (PEED) framework and examines the associations among sensory, spatial, and social environmental dimensions, positive affect state, repetitive negative thinking, exercise enjoyment, psychological distress, and the Thermic Effect of Activity. Because the available instruments generally assessed only selected environmental or psychological domains rather than all constructs included in the PEED framework, the Prenatal Exercise Environment and Experience Questionnaire (PEEQ) was newly developed for the present study as a framework-based composite questionnaire, with selected psychological items informed by or adapted from established measures.

The study extends previous research in three respects. First, it conceptualizes the prenatal exercise environment as a multidimensional construct rather than as a single background condition. Second, it examines positive affect state and repetitive negative thinking as parallel psychological pathways within the same framework. Third, it combines theory-driven structural equation modeling with exploratory machine learning analyses to examine both hypothesized structural associations and potential non-linear patterns in activity-related outcomes.

The proposed PEED framework was newly developed for the present study as an integrative conceptual framework rather than being directly adapted from a single established theory. It was informed by environmental psychology, evidence on affective responses to exercise, cognitive appraisal perspectives, and research on repetitive negative thinking. The framework proposes two parallel psychological pathways through which perceptions of the prenatal exercise environment may be associated with exercise experience. The affective pathway links more favorable environmental perceptions with a more positive affective state and, subsequently, greater exercise enjoyment. The cognitive pathway links more favorable environmental perceptions with lower repetitive negative thinking and, in turn, greater exercise enjoyment. In addition to these indirect pathways, favorable environmental perceptions may also be directly associated with greater exercise enjoyment by improving perceived comfort, safety, accessibility, and support. Exercise enjoyment is further hypothesized to be inversely associated with psychological distress.

Accordingly, this study aimed to examine the associations among the perceived prenatal exercise environment, positive affective state (AFF), repetitive negative thinking, exercise enjoyment, and psychological distress within the proposed PEED framework. The following hypotheses were tested in the structural model:

*H1*: More favorable perceptions of the prenatal exercise environment are positively associated with a more positive affective state.

*H2*: More favorable perceptions of the prenatal exercise environment are negatively associated with repetitive negative thinking.

*H3*: A more positive affective state is positively associated with exercise enjoyment.

*H4*: Repetitive negative thinking is negatively associated with exercise enjoyment.

*H5*: Exercise enjoyment is negatively associated with psychological distress.

*H6*: More favorable perceptions of the prenatal exercise environment are directly and positively associated with exercise enjoyment.

In addition to testing these theory-derived hypotheses, exploratory machine learning analyses were conducted to identify the relative importance of environmental, affective, cognitive, and background variables in predicting the Thermic Effect of Activity and to explore potential non-linear patterns.

## 2 Materials and methods

### 2.1 Research design and subjects

This study used a cross-sectional online survey design. Five medical centers participated as collaborating questionnaire-dissemination sites, with one center located in each of Sichuan, Hubei, Shanxi, Chongqing, and Guangdong. The centers were selected using convenience-based site selection. All five centers initially considered participated in the study, and no additional centers were screened or excluded. Data was collected through Wenjuanxing in October 2025. Staff at the collaborating centers provided potentially eligible pregnant women with information about the study and access to a common online questionnaire link. Eligible participants were recruited using convenience sampling, and participation was voluntary. Because the same questionnaire link was used across all collaborating centers and no site-specific identifier was collected, individual responses could not be reliably attributed to a particular medical center. Eligibility was assessed according to the predefined inclusion and exclusion criteria, and no randomization, quota sampling, or additional selection procedures were applied. All participants had to be 18 or older, physically capable of completing an online questionnaire on their own and had reported exercising at least once while pregnant to be eligible. Exclusion criteria included physician-diagnosed contraindications to exercise, multiple pregnancy, or incomplete questionnaire responses. All subjects granted informed permission before engaging in the study. The research procedure received approval from the institutional ethical committee (Approval No. EC-2025-097) and was performed in compliance with the Declaration of Helsinki (17).

### 2.2 Conceptual framework

All analyses were guided by the Prenatal Exercise Environment Dual-pathway (PEED) framework (Figure 1), which conceptualizes the exercise environment as a multidimensional construct influencing behavioral outcomes through parallel affective and cognitive pathways. Specifically, prenatal exercise environment was hypothesized to influence exercise enjoyment, perceived exertion, and psychological outcomes through positive affect and repetitive negative thinking.

**Figure 1:**
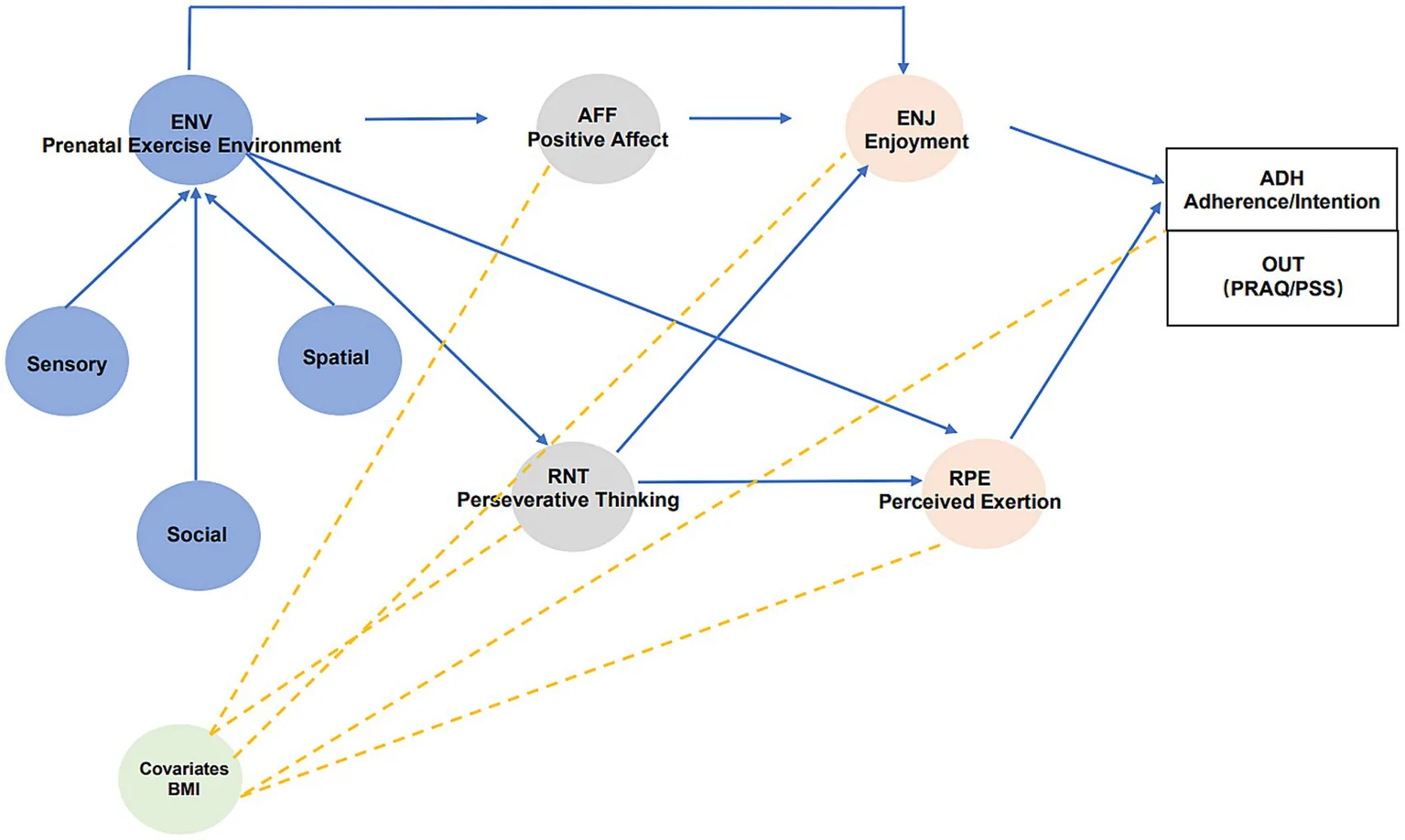
Prenatal Exercise Environment Dual-pathway (PEED) conceptual framework. The figure illustrates the proposed relationships among ENV, AFF, RNT, ENJ, RPE, ADH, and OUT. ENV is conceptualized as comprising sensory, spatial, and social environmental dimensions. Solid blue arrows represent the proposed relationships among the conceptual framework variables, whereas dashed orange lines indicate proposed covariate relationships involving BMI. This figure represents the broader conceptual framework rather than the final structural model tested in the PLS-SEM analysis.

### 2.3 Measures

#### 2.3.1 Prenatal exercise environment and experience questionnaire

The PEEQ was newly developed for the present study as a framework-based composite questionnaire guided by the PEED framework and relevant literature on prenatal exercise, environmental psychology, and exercise behavior. All environmental items were newly generated for the present study. The AFF items were researcher-developed and were conceptually organized to provide brief coverage of positive and negative affect with differing levels of activation, consistent with the valence–arousal framework of the circumplex model of affect (18); no validated affect instrument was directly administered or adapted. The ENJ items were researcher-developed for the present study and were not directly adapted from a validated exercise-enjoyment instrument. The RNT items were adapted and contextualized from the conceptual and item framework of the Perseverative Thinking Questionnaire (PTQ) to the prenatal-exercise context (Available under CC BY 3.0) (19). Within the OUT construct, P1 and P3 were researcher-developed items informed by pregnancy-related anxiety concepts represented in the Pregnancy-Related Anxiety Questionnaire (PRAQ) (20), whereas P2 was a researcher-developed item informed by the perceived-control-over-stress concept represented in the Perceived Stress Scale (PSS) (21). Neither the PRAQ nor the PSS was administered as a complete standardized instrument. For the RNT component, the wording and response format were modified and contextualized for thoughts occurring during prenatal exercise, and the original PTQ scoring procedure was not used. Because the OUT items were researcher-developed rather than directly adapted from PRAQ or PSS items, the original wording, response formats, and scoring procedures of the PRAQ and PSS were not reproduced. The preliminary questionnaire was reviewed by three experts in prenatal exercise, exercise psychology, and maternal health as a preliminary qualitative assessment of item relevance, clarity, and coverage of the proposed constructs. No formal quantitative content-validity indices, such as the CVI, I-CVI, S-CVI, or CVR, were calculated. The questionnaire was subsequently pilot tested in 10 pregnant women to assess comprehensibility and feasibility. Based on expert and participant feedback, minor wording revisions were made to improve item clarity and comprehensibility; no items were added or removed at this stage. Minor wording revisions were made before the formal survey. Exploratory factor analysis was used to examine the underlying factor structure. The measurement model was subsequently evaluated using partial least squares structural equation modeling (PLS-SEM), including indicator loadings, Cronbach’s alpha, composite reliability, average variance extracted (AVE), the Fornell–Larcker criterion, and the heterotrait–monotrait ratio (HTMT) to assess indicator reliability, internal consistency reliability, convergent validity, and discriminant validity. In addition, a supplementary covariance-based structural equation modeling (CB-SEM) measurement model was estimated as a same-sample confirmatory assessment of the seven-factor first-order measurement structure identified through the preceding EFA and the theoretically specified PEED framework. The model comprised ENV1, ENV2, ENV3, AFF, ENJ, RNT, and OUT, and overall fit was evaluated using χ^2^/df, root mean square error of approximation (RMSEA), goodness-of-fit index (GFI), Tucker–Lewis index (TLI), and comparative fit index (CFI). This analysis was conducted as a supplementary assessment of whether the EFA-derived structure showed acceptable fit under a covariance-based modeling framework, rather than as an independent validation. The structural relationships specified in the PEED framework were subsequently evaluated using PLS-SEM with Bootstrapping.

The final PEEQ measurement model comprised 34 retained items across seven first-order constructs: Physical and Sensory Environment (ENV1, 11 items), Spatial and Privacy Environment (ENV2, 3 items), Social and Organizational Support (ENV3, 3 items), positive affective state (AFF, 6 items), Exercise Enjoyment (ENJ, 3 items), Repetitive Negative Thinking (RNT, 5 items), and Psychological Distress (OUT, 3 items). Representative items included “Air was well-ventilated” (ENV1), “The place was not crowded and had enough space to move” (ENV2), “My peers or family encouraged me to continue” (ENV3), “Excited” and “Nervous” (AFF), “I enjoyed this exercise” (ENJ), “I kept thinking about worries or negative thoughts” (RNT), and “I worry that something may go wrong during pregnancy” (OUT).

ENV1, ENV2, ENV3, ENJ, and OUT items were rated on 7-point Likert scales ranging from 1 (strongly disagree) to 7 (strongly agree). AFF items were rated from 1 (very slightly) to 7 (very strongly), whereas RNT items were rated from 1 (never) to 7 (very often). Construct scores were calculated as the mean of the corresponding retained items. The three negatively worded AFF items (A4–A6) were reverse coded using 8 minus the original response, such that higher AFF scores represented a more positive affective state. The positively worded stress-control item P2 within OUT was similarly reverse-coded before calculation of the OUT score. Higher scores therefore represented more favorable environmental perceptions for ENV1–ENV3, more positive affect for AFF, greater exercise enjoyment for ENJ, more frequent repetitive negative thinking for RNT, and greater psychological distress for OUT.

The complete wording, construct assignment, item provenance, response options, reverse-coding information, and scoring procedures for all 34 retained PEEQ items are provided in Supplementary Table S3. Perceived exertion and the additional exercise-adherence/intention questions were administered as separate study measures and were not indicators of the final seven-factor PEEQ measurement model. The separately administered exercise-behavior/adherence/intention questions were researcher-developed for descriptive assessment and were not combined into psychometric constructs or composite scores. They assessed exercise type, weekly exercise frequency, session duration, interruption of exercise for ≥1 week during the previous 4 weeks (No/Yes), plans to continue exercising during the next 2 weeks (Yes/No), and intention to continue exercising on a 0–10 scale. Exercise type, frequency, and duration contributed to the estimation of weekly TEA, whereas the interruption and intention-related items were not included in the subsequent SEM or machine-learning analyses. The complete wording, response options, and coding/scoring procedures for these six measures are provided in Supplementary Table S5.

#### 2.3.2 Anthropometric and background variables

Participants self-reported their body weight (kg) and height (cm). BMI was calculated using the standard formula: BMI = weight (kg)/height (m^2^) (22). Demographic and pregnancy-related variables included age (Age), trimester of pregnancy (ToP), first-time pregnancy status (PfT), and educational background (Edu).

The Thermic Effect of Activity (TEA) was estimated based on self-reported exercise characteristics. Perceived exertion was assessed separately from the PEEQ measurement model using a study-specific grouped presentation based on the Borg 6–20 RPE framework. The response categories were 6 (very, very light), 7–8 (very light), 9–10 (light), 11–12 (somewhat hard), 13–14 (hard), 15–16 (very hard), 17–18 (extremely hard), and 19–20 (maximal effort). A non-commercial academic license for use of the Borg RPE materials in English and Simplified Chinese was subsequently obtained from BorgPerception AB. MET values were assigned according to the 2024 Adult Compendium of Physical Activities by matching the reported exercise type and intensity with the corresponding Compendium activity, with Borg RPE used to support intensity classification rather than to generate MET values independently (23). In the present study, TEA was operationalized as estimated gross weekly exercise-related energy expenditure and was expressed in kcal/week using the following formula:


$$\begin{array}{l} \text{Weekly}\;\mathrm{TEA}\;(\text{kcal}/\text{week})=\text{body weight}\;(\mathrm{kg})\\ \times \text{METs}\times \text{exercise duration}\;(\text{hours}/\text{session})\\ \times \text{weekly exercise frequency}\;(\text{sessions}/\text{week}) \end{array}$$

### 2.4 Sample size and power analysis

Prior to participant recruitment, *a priori* sample-size calculation was conducted using G*Power version 3.1.9.7. The calculation was based on an ANCOVA fixed-effects model for main effects and interactions, assuming a medium effect size of *f* = 0.25, α = 0.05, statistical power (1 − β) = 0.95, three groups, a numerator degree of freedom of 2, and one covariate. The minimum required total sample size was estimated to be 251 participants (actual power = 0.951). A total of 368 questionnaires were subsequently received. Seven responses were excluded because the participants reported having been advised by a physician to avoid exercise during pregnancy, resulting in a final analytical sample of 361 participants, which exceeded the minimum sample-size requirement.

### 2.5 Statistical analysis

All statistical analyses were conducted using SPSSAU, Origin 2024, and SmartPLS 4 and R version 4.5.2. EFA was conducted in SPSSAU using the principal component method for extraction with Varimax rotation. The number of factors was determined using the Kaiser criterion (eigenvalues > 1). A scree plot was additionally generated, whereas parallel analysis was not performed.

The Shapiro–Wilk test was used to assess the normality of the continuous variables. Because the continuous study variables did not meet the assumption of normality, Spearman’s rank correlation analysis was used to examine associations among the study variables. Differences across gestational trimesters were assessed using the Kruskal–Wallis H test because the study variables did not meet the assumption of normality. Where a statistically significant overall difference was identified, *post hoc* pairwise comparisons with adjustment for multiple testing were planned. Bar charts were generated using Origin 2024 for visualization.

SEM was conducted using SmartPLS 4 to evaluate the hypothesized PEED framework. The measurement model was assessed using indicator loadings, composite reliability, average variance extracted, and discriminant validity. The structural model was evaluated using collinearity diagnostics, standardized path coefficients, and coefficients of determination (R^2^). Bootstrapping with 5,000 resamples was performed to assess the statistical significance of the path coefficients.

Harman’s single-factor test was conducted to assess potential common method bias arising from the use of self-reported data collected at a single time point. The variance explained by the first unrotated factor was compared with the predefined 40% criterion.

Machine-learning analyses were conducted in R version 4.5.2 using the rpart, rpart.plot, and randomForest packages. Following reverse coding of the three negatively worded AFF items, the revised positive affective state score was used in all machine-learning analyses. The candidate predictors of weekly TEA included Age, ToP, PfT, Edu, BMI, ENV1, ENV2, ENV3, AFF, ENJ, RNT, and OUT. Using a fixed random seed (set.seed(123)), the dataset was randomly partitioned into a training set comprising approximately 70% of the observations (n = 252) and an independent test set comprising the remaining 30% (n = 109). The same partition and predictor set were used for both models.

A regression tree was fitted to the training dataset using recursive binary partitioning with TEA as the continuous outcome. The initial tree was grown using a complexity parameter of 0.001, a minimum split size of 20, a minimum terminal-node size of 7, and a maximum tree depth of 30. Ten-fold internal cross-validation was used to evaluate tree complexity, and the final model was pruned using the complexity parameter corresponding to the minimum cross-validated error (cp = 0.0107).

The Random Forest regression model was trained using 500 trees, with four candidate predictors considered at each split and a terminal-node size of five. No exhaustive grid-search procedure was conducted; these parameters were prespecified or retained at their standard regression settings. Internal model evaluation was performed using out-of-bag observations and 10-fold cross-validation within the training dataset. For the cross-validation analysis, predictions for each held-out fold were combined to calculate pooled out-of-fold performance.

Both models were externally evaluated using the independent test dataset. Predictive performance was assessed using the coefficient of determination (R^2^), root mean square error (RMSE), mean absolute error (MAE), and mean squared error (MSE). Random Forest predictor importance was quantified using the permutation-based percentage increase in mean squared error (%IncMSE) calculated from the out-of-bag observations. Observed and predicted TEA values were plotted to visually assess prediction accuracy.

Although BMI and other demographic characteristics (e.g., gestational trimester, educational level, and pregnancy history) were considered during model development, they were not included as covariates in the final SEM because the aim of the model was to test the hypothesized relationships among the latent constructs of the PEED framework.

## 3 Results

### 3.1 Demographic characteristics

Regarding gestational distribution, 189 (52.35%) were in the first trimester, 116 (32.13%) in the second trimester, and 56 (15.52%) in the third trimester in Table 1.

**Table 1:** Demographic and pregnancy-related characteristics of the study participants (*n* = 361).

Variable	*n* (%) or median (Q1–Q3)
Chinese citizen	361 (100%)
Age (years), median (Q1–Q3)	29.00 (25.00–33.00)
20–25	105 (29.09%)
26–34	203 (56.23%)
35+	53 (14.68%)
BMI (kg/m^2^), median (Q1–Q3)	20.20 (18.13–21.94)
Underweight	102 (28.25%)
Normal weight	244 (67.59%)
Overweight	15 (4.16%)
Trimester of gestation	
First trimester	189 (52.35%)
Second trimester	116 (32.13%)
Third trimester	56 (15.52%)
Highest educational level	
Below Bachelor	149 (41.27%)
Bachelor’s degree	171 (47.37%)
Postgraduate or above	41 (11.36%)
Pregnancy for the first time	
Yes	231 (63.98%)
No	130 (36.02%)

Continuous variables are presented as median (Q1–Q3), and categorical variables are presented as n (%). BMI, body mass index; Q1, first quartile; Q3, third quartile.

### 3.2 Measurement and structural model results

An initial EFA was conducted on 35 candidate items. Sampling adequacy was high (KMO = 0.917), and Bartlett’s test of sphericity was significant (χ^2^ = 6654.509, df = 595, *p* < 0.001). Seven factors with eigenvalues greater than 1 were extracted, accounting for 66.364% of the rotated cumulative variance. The complete rotated EFA loading matrix, including cross-loadings and communalities, is provided in Supplementary Table S4. One item (S4) showed a very low communality (0.093) and no meaningful loading on the extracted factors and was therefore removed. The remaining 34 items were retained for subsequent measurement-model evaluation. The measurement model demonstrated satisfactory psychometric properties for the seven first-order reflective constructs. Cronbach’s alpha values ranged from 0.809 to 0.942, composite reliability (rho_C) values ranged from 0.886 to 0.950, and average variance extracted (AVE) values ranged from 0.632 to 0.733, exceeding the recommended thresholds of 0.70, 0.70, and 0.50, respectively. Indicator loadings were above 0.70, supporting indicator reliability, internal consistency reliability, and convergent validity. Discriminant validity among the first-order constructs was also supported. The square root of the AVE for each first-order construct exceeded its correlations with the other first-order constructs, and all corresponding HTMT values were below 0.85. The higher-order environmental construct (ENV), formed by ENV1, ENV2, and ENV3, was evaluated separately from the first-order reflective constructs. Detailed construct-level measurement results and the corresponding HTMT matrix are provided in Supplementary Table S2.

As a same-sample confirmatory assessment of the first-order PEEQ measurement structure, a seven-factor CB-SEM measurement model comprising ENV1, ENV2, ENV3, AFF, ENJ, RNT, and OUT was estimated. The model demonstrated good overall fit (χ^2^ = 576.941, df = 506, χ^2^/df = 1.140, RMSEA = 0.020, 90% CI [0.009, 0.027], GFI = 0.918, TLI = 0.988, and CFI = 0.989), providing additional preliminary support for the measurement structure of the PEEQ.

For the PLS-SEM structural model, the saturated and estimated models yielded SRMR values of 0.105 and 0.118, respectively. The estimated-model SRMR indicated limited approximate overall model fit and was therefore considered when interpreting the structural-model findings. Inner-model VIF values ranged from 1.000 to 1.211, indicating that collinearity was not a critical concern.

The structural-model results are presented in Table 2 and Figure 2. The perceived prenatal exercise environment was positively associated with positive affective state (β = 0.328, t = 7.081, *p* < 0.001, 95% CI [0.238, 0.419]) and negatively associated with repetitive negative thinking (β = −0.297, t = 6.568, *p* < 0.001, 95% CI [−0.387, −0.211]). Positive affective state was positively associated with exercise enjoyment (β = 0.143, t = 2.681, *p* = 0.007, 95% CI [0.042, 0.249]), whereas repetitive negative thinking was negatively associated with exercise enjoyment (β = −0.216, t = 4.599, *p* < 0.001, 95% CI [−0.309, −0.127]). Exercise enjoyment was negatively associated with psychological distress (β = −0.253, t = 5.454, *p* < 0.001, 95% CI [−0.348, −0.165]). The prenatal exercise environment was also directly and positively associated with exercise enjoyment (β = 0.270, t = 5.232, *p* < 0.001, 95% CI [0.167, 0.369]). Thus, H1–H6 were statistically supported.

**Table 2:** PLS-SEM structural-path estimates, hypothesis-testing results, and model-evaluation indices.

A. Structural-path and hypothesis-testing results							
Hypothesis	Structural path	β	t	*p*	95% CI	Inner VIF	Decision
H1	ENV → AFF	0.328	7.081	<0.001	[0.238, 0.419]	1.000	Supported
H2	ENV → RNT	−0.297	6.568	<0.001	[−0.387, −0.211]	1.000	Supported
H3	AFF → ENJ	0.143	2.681	0.007	[0.042, 0.249]	1.178	Supported
H4	RNT → ENJ	−0.216	4.599	<0.001	[−0.309, −0.127]	1.153	Supported
H5	ENJ → OUT	−0.253	5.454	<0.001	[−0.348, −0.165]	1.000	Supported
H6	ENV → ENJ	0.270	5.232	<0.001	[0.167, 0.369]	1.178	Supported

B. Model-evaluation indices and explained variance	
Model-evaluation index	Value
SRMR, saturated model	0.105
SRMR, estimated model	0.118
Inner-model VIF range	1.000–1.211
R^2^ for AFF	0.108
R^2^ for RNT	0.088
R^2^ for ENJ	0.218
R^2^ for OUT	0.064

β, standardized path coefficient; CI, confidence interval; VIF, variance inflation factor; SRMR, standardized root mean square residual; R^2^, coefficient of determination; ENV, prenatal exercise environment; AFF, positive affective state; RNT, repetitive negative thinking; ENJ, exercise enjoyment; OUT, psychological distress. Bootstrap results were based on 5,000 resamples.

**Figure 2:**
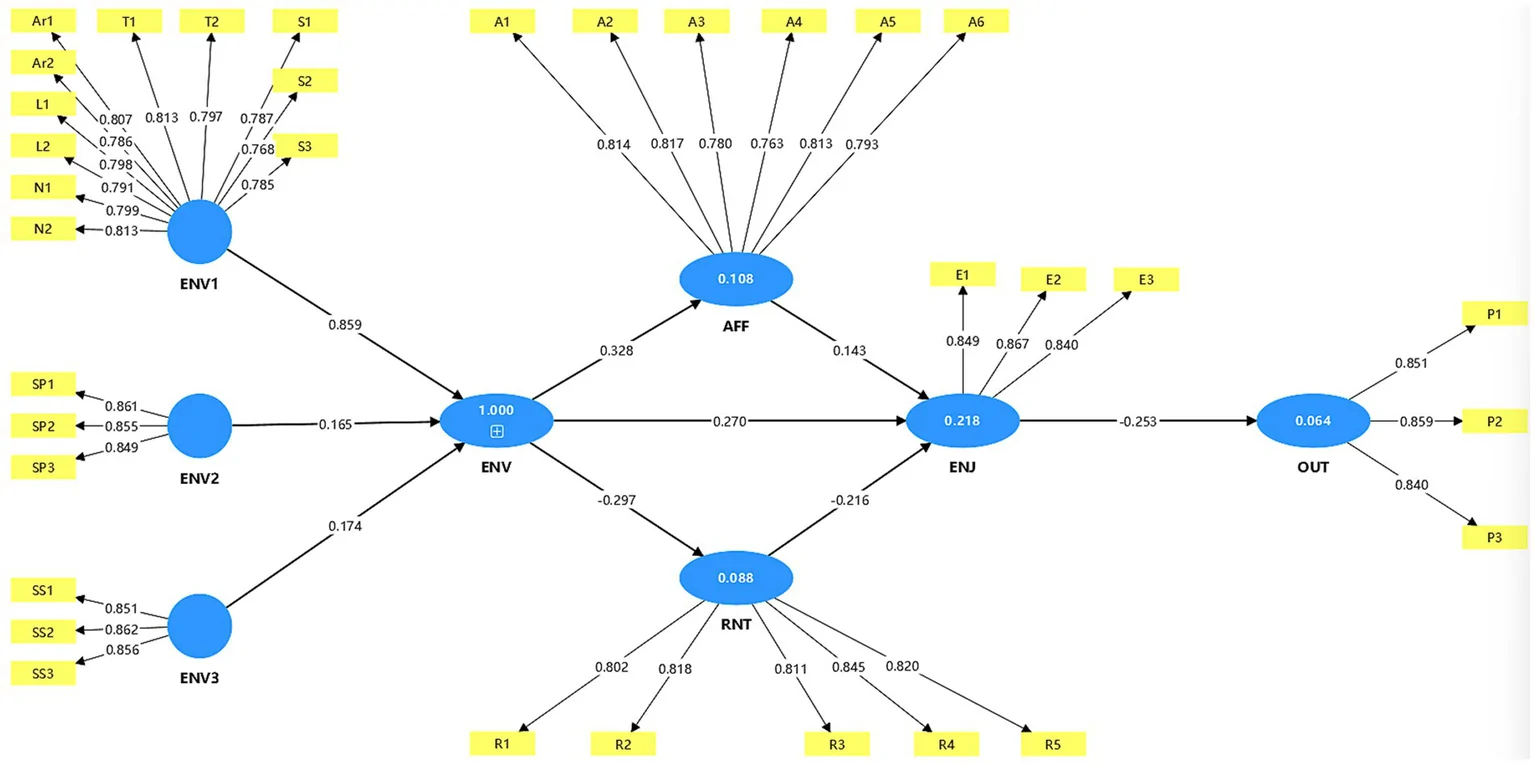
PLS-SEM structural model of the PEED framework. Structural-path values represent standardized coefficients (*β*), values within endogenous constructs represent *R*^2^, and values adjacent to item indicators represent indicator loadings. ENV1, ENV2, and ENV3 represent the three first-order dimensions of ENV.

The model explained 10.8% of the variance in positive affective state (R^2^ = 0.108), 8.8% in repetitive negative thinking (R^2^ = 0.088), 21.8% in exercise enjoyment (R^2^ = 0.218), and 6.4% in psychological distress (R^2^ = 0.064). The relatively low explained variance for psychological distress, together with the elevated estimated-model SRMR, indicates that the model provides only a partial account of psychological distress and that other relevant clinical, social, psychological, and contextual determinants may not have been captured by the present framework. No additional items were removed during the PLS-SEM measurement-model assessment.

### 3.3 Comparison across gestational trimesters

Because the continuous study variables did not meet the assumption of normality, the Kruskal–Wallis H test was used to examine differences across gestational trimesters. As shown in Table 3, no statistically significant differences were detected for ENV1 (H = 0.508, *p* = 0.776), ENV2 (H = 2.821, *p* = 0.244), ENV3 (H = 4.683, *p* = 0.096), positive affective state (AFF; H = 1.645, *p* = 0.439), ENJ (H = 4.318, *p* = 0.115), RNT (H = 0.577, *p* = 0.749), or OUT (H = 2.963, *p* = 0.227). No *post hoc* pairwise comparisons were therefore conducted. These findings indicate that no statistically significant trimester-related differences were detected for the examined variables; however, the non-significant results should not be interpreted as evidence of equivalence among the trimester groups.

**Table 3:** Comparison of study variables across gestational trimesters using the Kruskal–Wallis H test.

Variable	First trimester (*n* = 189), Median (Q1–Q3)	Second trimester (n = 116), Median (Q1–Q3)	Third trimester (*n* = 56), Median (Q1–Q3)	H	*p*
ENV1	4.333 (3.4–5.4)	4.333 (3.3–5.3)	4.458 (3.4–5.4)	0.508	0.776
ENV2	4.333 (3.0–5.3)	4.667 (3.3–5.7)	4.333 (3.3–5.3)	2.821	0.244
ENV3	4.667 (3.3–5.7)	4.333 (3.3–5.7)	4.000 (3.0–4.9)	4.683	0.096
AFF	4.333 (3.3–5.3)	4.333 (3.3–5.3)	3.917 (2.7–5.3)	1.645	0.439
ENJ	4.333 (3.3–5.7)	4.333 (3.1–5.7)	4.167 (3.0–5.0)	4.318	0.115
RNT	3.800 (2.6–4.8)	3.800 (2.8–4.4)	3.800 (2.8–5.0)	0.577	0.749
OUT	3.667 (2.7–4.7)	3.333 (2.7–4.6)	4.000 (2.8–5.3)	2.963	0.227

Values are presented as median (Q1–Q3). ENV1, physical and sensory environment; ENV2, spatial and privacy environment; ENV3, social and organizational support; AFF, positive affective state; ENJ, exercise enjoyment; RNT, repetitive negative thinking; OUT, psychological distress.

### 3.4 Normality and correlation analysis

Prior to the correlation analysis, the normality of the continuous variables was assessed using the Shapiro–Wilk test. Age, BMI, ENV1, ENV2, ENV3, AFF, ENJ, RNT, OUT, and TEA did not meet the assumption of normality (all *p* < 0.05). Accordingly, Spearman’s rank correlation analysis was conducted. The overall correlation patterns are presented in Figure 3, and the complete correlation coefficients and corresponding significance levels are reported in Supplementary Table S1.

**Figure 3:**
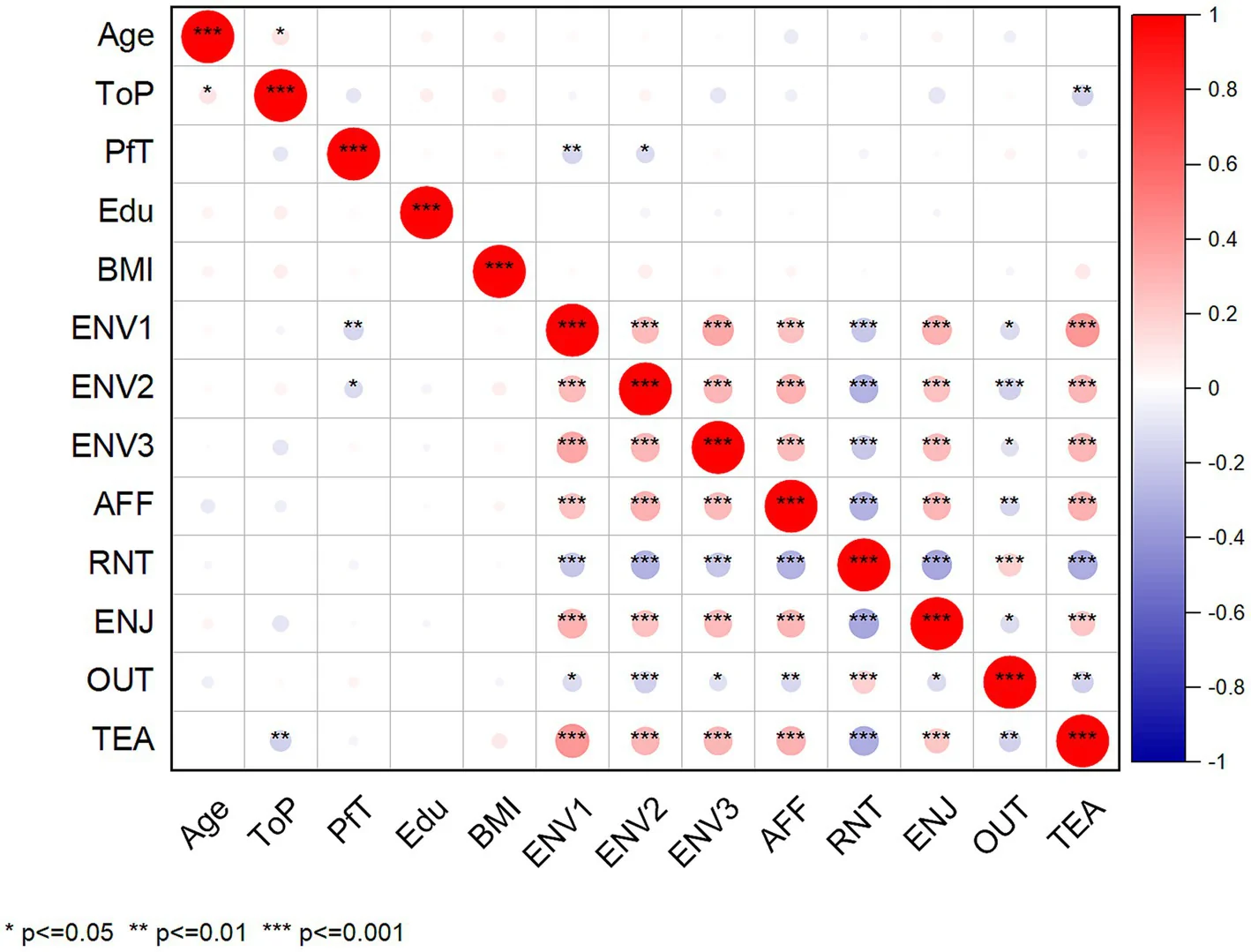
Spearman correlation matrix of the study variables. Circle color indicates the direction of the correlation, with red representing positive correlations and blue representing negative correlations, while circle size reflects the absolute magnitude of the correlation coefficient (*ρ*). Statistical significance is indicated by *p* ≤ 0.05*, *p* ≤ 0.01**, and *p* ≤ 0.001***.

All three exercise-environment dimensions were positively correlated with positive affective state (ENV1: ρ = 0.242; ENV2: ρ = 0.318; ENV3: ρ = 0.271; all *p* < 0.001) and exercise enjoyment (ENV1: ρ = 0.314; ENV2: ρ = 0.254; ENV3: ρ = 0.279; all *p* < 0.001). Conversely, ENV1, ENV2, and ENV3 were negatively correlated with repetitive negative thinking (ρ = −0.218, −0.296, and −0.217, respectively; all *p* < 0.001).

Positive affective state was positively correlated with exercise enjoyment (ρ = 0.281, *p* < 0.001) and negatively correlated with repetitive negative thinking (ρ = −0.293, *p* < 0.001) and psychological distress (ρ = −0.141, *p* < 0.01). Exercise enjoyment was negatively correlated with repetitive negative thinking (ρ = −0.329, *p* < 0.001) and psychological distress (ρ = −0.129, *p* < 0.05), whereas repetitive negative thinking was positively correlated with psychological distress (ρ = 0.192, *p* < 0.001).

Weekly TEA was positively correlated with ENV1 (ρ = 0.411, *p* < 0.001), ENV2 (ρ = 0.288, *p* < 0.001), ENV3 (ρ = 0.297, *p* < 0.001), positive affective state (ρ = 0.309, *p* < 0.001), and exercise enjoyment (ρ = 0.224, *p* < 0.001). In contrast, weekly TEA was negatively correlated with repetitive negative thinking (ρ = −0.316, *p* < 0.001) and psychological distress (ρ = −0.170, *p* < 0.01). Associations involving most demographic and pregnancy-related variables were generally weak.

### 3.5 Machine learning analysis for TEA prediction

To further identify the key predictors of weekly TEA and explore potential nonlinear and hierarchical patterns among the study variables, regression-tree and Random Forest analyses were conducted using the revised AFF score after reverse coding of the three negatively worded items. The pruned regression tree is presented in Figure 4a. ENV1 emerged as the primary splitting variable at a threshold of 5.5. Participants with ENV1 scores below 5.5 constituted 80% of the training sample and had a predicted mean TEA of 438 kcal/week. Among participants with ENV1 scores of 5.5 or higher, RNT provided subsequent splits at 2.3 and 3.9. Within the subgroup with RNT scores below 2.3, AFF further differentiated TEA at a threshold of 5.5: participants with AFF scores below 5.5 had a predicted mean TEA of 1,447 kcal/week, whereas those with AFF scores of 5.5 or higher had the highest predicted mean TEA of 3,165 kcal/week. No separate inferential interaction test was conducted. Therefore, these findings represent an exploratory sequential splitting pattern rather than a formally tested statistical interaction.

**Figure 4:**
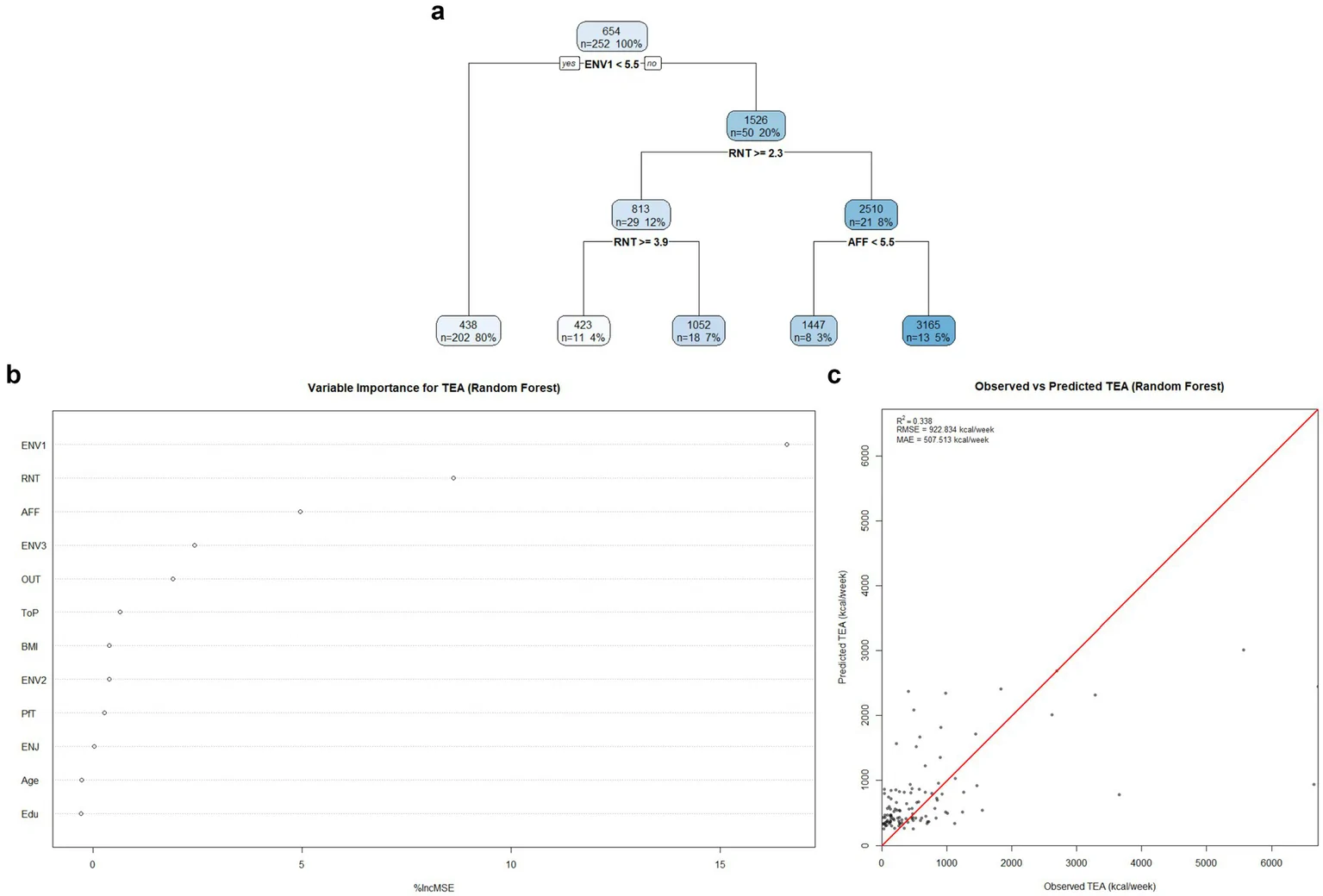
**(a)** Pruned regression tree for weekly TEA prediction. The tree was developed using the training dataset (*n* = 252). Internal nodes show the mean predicted TEA for each subgroup, together with the subgroup sample size and percentage of the training sample. Splits were based on ENV1, RNT, and AFF, and terminal nodes represent the predicted mean weekly TEA (kcal/week) for the corresponding subgroups. **(b)** Random Forest variable importance for weekly TEA prediction. Predictor importance is expressed as the permutation-based percentage increase in mean squared error (%IncMSE), with larger positive values indicating greater predictive contribution. Negative importance values indicate that permutation of the corresponding predictor did not increase prediction error and should not be interpreted as negative associations with TEA. **(c)** Observed versus Random Forest-predicted weekly TEA. Each point represents one participant in the independent test dataset. The red diagonal line indicates perfect agreement between observed and predicted values. Model performance in the test set was *R*^2^ = 0.338, RMSE = 922.834 kcal/week, and MAE = 507.513 kcal/week.

The Random Forest analysis further evaluated the relative importance of the 12 candidate predictors using the scaled permutation-based percentage increase in out-of-bag mean squared error (%IncMSE; Figure 4b). ENV1 showed the highest importance (%IncMSE = 16.595), followed by RNT (8.612), AFF (4.959), ENV3 (2.422), OUT (1.907), ToP (0.638), BMI (0.380), ENV2 (0.378), PfT (0.269), ENJ (0.026), Age (−0.270), and Edu (−0.293). The negative importance values for Age and Edu indicate that permuting these variables did not increase the out-of-bag prediction error and that they did not provide stable incremental predictive information. These negative values should not be interpreted as evidence of negative associations with TEA.

Internal evaluation of the Random Forest model using out-of-bag observations yielded a mean squared residual of 435,527.9 and explained 40.9% of the variance in TEA. Ten-fold cross-validation within the training dataset produced pooled out-of-fold predictions with an R^2^ of 0.350, an RMSE of 691.895 kcal/week, and an MAE of 431.342 kcal/week. Across the 10 validation folds, the mean RMSE was 635.873 ± 288.223 kcal/week, and the mean MAE was 431.463 ± 158.500 kcal/week. Performance varied across folds, indicating that the model’s predictive generalizability should be interpreted cautiously.

In the independent test set (*n* = 109), the pruned regression tree achieved an R^2^ of 0.205, an RMSE of 1,011.591 kcal/week, and an MAE of 534.501 kcal/week. The Random Forest model demonstrated better test-set performance, with an R^2^ of 0.338, an RMSE of 922.834 kcal/week, an MAE of 507.513 kcal/week, and an MSE of 851,623.360 (kcal/week)^2^. As shown in Figure 4c, the Random Forest model captured part of the variation in weekly TEA; however, prediction errors were greater at higher observed TEA values, with a tendency to underestimate extreme values.

## 4 Discussion

In a sample of 361 pregnant women, the present study used the proposed PEED framework to examine associations among perceptions of the prenatal exercise environment, positive affective state, repetitive negative thinking, exercise enjoyment, psychological distress, and weekly TEA. The structural-model results indicated that more favorable environmental perceptions were associated with a more positive affective state, lower repetitive negative thinking, and greater exercise enjoyment. Positive affective state and repetitive negative thinking were, in turn, associated with exercise enjoyment, whereas exercise enjoyment was inversely associated with psychological distress. The machine-learning analyses additionally identified the sensory and physical environment as the strongest predictor of weekly TEA, followed by repetitive negative thinking and positive affective state. These findings provide preliminary structural and predictive support for the PEED framework but should be interpreted as associations rather than definitive mechanisms. Given the cross-sectional design, directional and causal inferences remain tentative.

The positive association between perceptions of the prenatal exercise environment and positive affective state extends previous prenatal physical-activity research that has primarily examined individual, interpersonal, and contextual barriers and facilitators. Studies among pregnant women have identified physical discomfort, perceived safety, social support, and self-efficacy as relevant to physical activity behavior (4–7, 9). Environmental research during pregnancy has also examined broader exposures such as greenspace and walkability in relation to objectively assessed physical activity (8). However, such studies differ from the present work because they largely concern neighborhood-level exposure rather than pregnant women’s perceptions of the immediate exercise setting. The present study therefore extends this literature by considering sensory and physical characteristics, spatial comfort and privacy, and social and organizational support together as a multidimensional exercise environment. Although broader environmental-psychology research supports associations between environmental characteristics and emotional appraisal (10–12), the present cross-sectional data cannot establish temporal direction. In broader exercise research, affective responses to exercise have also been associated with subsequent motivation and physical activity behavior (16); however, the relatively modest AFF–ENJ association observed here suggests that positive affect represents only one component of exercise enjoyment during pregnancy.

The associations of the prenatal exercise environment with repetitive negative thinking, and of repetitive negative thinking with exercise enjoyment, address a comparatively underexplored area of prenatal exercise research. Previous pregnancy-specific studies have focused primarily on barriers and facilitators of physical activity, behavioral correlates, and individual psychological factors such as self-efficacy (4–7, 9), whereas direct evidence linking perceived exercise environments with RNT during pregnancy remains limited. In the broader psychological literature, RNT has been described as a persistent cognitive process that can sustain worry and consume attentional resources (13, 14), while pregnancy-specific evidence has more generally linked prenatal physical activity with maternal mental health (15). The absolute coefficient for the RNT–ENJ association was numerically larger than that for the AFF–ENJ association (|β| = 0.216 versus 0.143). However, these two coefficients were not formally compared, and the difference should not be interpreted as demonstrating the dominance of the cognitive pathway. Measurement characteristics shared self-report methods, and unmeasured psychological factors may also have contributed to the observed pattern. Thus, although the results are consistent with dual-process perspectives, the relative roles of the affective and cognitive pathways require further validation through longitudinal or experimental designs (24–26).

Exercise enjoyment was inversely associated with psychological distress. Previous pregnancy-specific studies and reviews have linked greater prenatal physical activity or participation in structured exercise with more favorable maternal mental-health outcomes (1, 15, 27). However, the present finding differs conceptually from this literature because it concerns the subjective experience of exercise enjoyment rather than physical activity or exercise participation itself. It therefore identifies a potentially relevant experiential correlate of maternal psychological status but does not establish that greater enjoyment reduces psychological distress. Reverse directionality is plausible, as women experiencing less psychological distress may perceive exercise as more enjoyable. In addition, shared self-report methods may introduce common method bias and inflate associations among psychological constructs (28). Therefore, the ENJ–OUT relationship should be viewed as associative rather than strictly protective. Moreover, the model explained only 6.4% of the variance in psychological distress. Together with the elevated estimated-model SRMR, this limited explanatory power indicates that important determinants of maternal psychological distress were not included in the model. These may include previous mental health conditions, sleep, socioeconomic pressures, pregnancy complications, relationship factors, and stressful life events. Accordingly, the significant ENJ–OUT association provides only a partial account of psychological distress and should not be interpreted as a comprehensive explanatory model.

No statistically significant differences in the examined variables were identified across gestational trimesters using the Kruskal–Wallis H test. However, the trimester groups were unevenly distributed, with 189 participants in the first trimester, 116 in the second trimester, and only 56 in the third trimester. The relatively small third-trimester subgroup may have reduced statistical power and precision, particularly for detecting modest between-group differences. In contrast, previous longitudinal research suggests that physical activity patterns may change over the course of pregnancy (29); therefore, the absence of statistically significant differences in the present cross-sectional sample should not be interpreted as evidence of equivalence or stability across gestational stages. More balanced trimester-specific samples and longitudinal follow-up are needed to examine within-person changes throughout pregnancy.

Taken together, the principal contribution of the present study is not simply the previously established association between prenatal physical activity and maternal psychological health (1, 15, 27). Rather, it extends prenatal exercise research by examining multidimensional perceptions of the immediate exercise environment together with affective state, repetitive negative thinking, exercise enjoyment, psychological distress, and an activity-related outcome within a single analytical framework. Previous pregnancy-specific studies have more often examined barriers, facilitators, psychological correlates, or broader environmental exposures separately (4–9). The PEED framework integrates these domains, while the complementary machine-learning analysis explores their relative predictive contributions. Given the cross-sectional design, these findings should be regarded as framework development and hypothesis generation rather than confirmation of causal mechanisms.

The machine-learning findings complement the structural-model results by identifying predictive patterns that were not specified as confirmatory hypotheses. In the Random Forest model, ENV1 showed the highest permutation importance, followed by RNT and AFF, whereas the remaining predictors provided smaller incremental predictive contributions. AFF emerged as the third-ranked predictor, indicating that affective factors should not be regarded as negligible in the prediction of TEA. The regression tree identified an exploratory sequential splitting pattern in which ENV1 was the primary splitting variable, followed by RNT and AFF within selected higher-ENV1 subgroups. No separate inferential interaction test was conducted; therefore, the observed pattern should not be interpreted as a statistically confirmed interaction or as evidence that the identified cut-off values represent established behavioral thresholds.

The Random Forest model achieved a test-set R^2^ of 0.338, and the observed-versus-predicted results showed greater errors and a tendency to underestimate very high TEA values. Thus, the model demonstrated partial predictive utility but did not provide highly accurate individual prediction across the full range of TEA. Regression-tree cut-off values may be sample-specific, and Random Forest importance reflects predictive contribution rather than causal influence. ENV1 should therefore be interpreted as the strongest predictor within the current model rather than as the dominant causal determinant of exercise behavior (30, 31). In addition, TEA was estimated using self-reported exercise type, frequency, duration, and perceived exertion, with MET values assigned from the Adult Compendium and RPE used to support intensity classification. Recall errors, activity misclassification, and measurement error in TEA may have influenced both model training and evaluation. Future research using objective activity measures, such as accelerometry or validated wearable devices, is needed to evaluate the reproducibility of these predictive patterns (32).

The observed intercorrelations among the ENV dimensions may reflect the integrated nature of perceived exercise environments, in which sensory, spatial, and social characteristics jointly contribute to the overall environmental experience. Nevertheless, overlap among the dimensions may make their independent contributions difficult to distinguish and may partly account for the prominence of ENV1. Although the higher-order environmental construct was used in the structural model, future research should continue to evaluate discriminant validity and consider whether higher-order modeling or dimensional refinement is appropriate in independent samples (10).

From a practical perspective, the findings suggest several preliminary considerations for prenatal exercise programs. Exercise settings could be reviewed for sensory comfort, including appropriate temperature, ventilation, lighting, noise levels, cleanliness, and equipment comfort. Programs may also consider privacy, perceived safety, accessibility, clear exercise instructions, and the availability of professional, family, or peer support. Pregnant women reporting persistent concerns or repetitive negative thinking may benefit from clearer safety information, opportunities to become gradually familiar with the exercise setting, and individualized communication from qualified prenatal exercise or healthcare professionals. However, these recommendations are hypothesis-generating. The present cross-sectional associations do not establish that modifying these environmental characteristics will increase exercise participation or reduce psychological distress. Their feasibility and effectiveness should be evaluated in prospective and randomized intervention studies. Recent evidence comparing in-person, remote, and blended lifestyle interventions among pregnant individuals with overweight or obesity further suggests that intervention effectiveness may vary according to delivery mode and intervention components, underscoring the importance of prospectively evaluating how environmental and contextual features can be incorporated into future prenatal exercise programs (33).

## 5 Limitations and future directions

The integration of SEM and machine learning bridges theory-driven and data-driven approaches. However, these methods rely on different analytical assumptions, and their convergence should be interpreted as complementary rather than confirmatory evidence (34). The cross-sectional design precludes determination of temporal order and does not permit causal conclusions regarding the proposed affective and cognitive pathways. Longitudinal and experimental studies are therefore required to examine whether changes in environmental perceptions precede changes in affective state, repetitive negative thinking, exercise enjoyment, psychological distress, or activity-related outcomes.

Several additional limitations should be acknowledged. First, the PEEQ was newly developed, and its psychometric evaluation was conducted within the same study sample. The present reliability and validity findings should therefore be regarded as preliminary. Independent validation is needed, including replication of the factor structure using confirmatory factor analysis in independent samples, test–retest reliability, measurement invariance across gestational stages and cultural groups, and criterion-related validity before broader application.

Second, recruitment relied on a voluntary online survey distributed through medical centers. Women who chose to participate may have been more interested in health or prenatal exercise than non-participants. This voluntary recruitment approach may therefore have introduced selection bias. In addition, eligibility required participants to have exercised at least once during pregnancy, meaning that women who were completely inactive were not represented. Individuals with limited internet access or digital literacy may also have been underrepresented. The self-administered format prevented independent verification of responses and may have introduced recall, social-desirability, and response biases.

Third, all participants were recruited from medical centers in selected regions of China. Cultural expectations, patterns of family support, exercise practices, socioeconomic conditions, and prenatal healthcare delivery may differ across countries and healthcare systems. The findings may therefore not be directly generalizable to pregnant women outside China, women not receiving care through medical centers, rural populations, or groups with different social and economic characteristics. Cross-cultural validation and replication in more diverse communities and healthcare settings are needed.

Fourth, TEA was an estimated gross weekly exercise-related energy-expenditure measure derived from self-reported exercise type, frequency, duration, and perceived exertion rather than an objective assessment of physical activity or adherence. It should not be interpreted as equivalent to objectively verified exercise adherence. Recall error, activity misclassification, and measurement error may therefore have affected TEA estimation and the subsequent predictive analyses. The relatively small third-trimester subgroup and the limited prediction of extreme TEA values may also reduce the precision and generalizability of the findings. Finally, the low explained variance for psychological distress and the limited approximate overall model fit indicates that the PEED framework does not capture all relevant determinants of maternal psychological health. Future studies should incorporate broader clinical, social, behavioral, and contextual variables, use objective activity measures, recruit more balanced and diverse samples, and prospectively test the proposed associations.

## 6 Conclusion

This cross-sectional study provides preliminary support for the proposed Prenatal Exercise Environment Dual-pathway framework. More favorable perceptions of the prenatal exercise environment were associated with a more positive affective state, lower repetitive negative thinking, and greater exercise enjoyment, while exercise enjoyment was inversely associated with psychological distress. The sensory and physical environment emerged as the strongest predictor of weekly TEA, followed by repetitive negative thinking and positive affective state; however, the predictive performance remained limited, and the regression-tree patterns should be interpreted as exploratory.

For prenatal exercise programs and healthcare practice, the findings suggest that exercise settings may benefit from attention to sensory comfort, privacy, perceived safety, accessibility, clear exercise guidance, and professional, family, or peer support. Healthcare and prenatal exercise professionals may also consider environmental preferences and persistent negative thinking when providing individualized exercise counseling. However, these implications are hypothesis-generating and require confirmation through longitudinal and intervention studies using independently validated measures and objective physical activity assessment.

## Data Availability

The raw data supporting the conclusions of this article will be made available by the authors, without undue reservation.
