# Supplementary material for: Associations between the prenatal exercise environment, exercise experience, and adherence: a cross-sectional study of affective and cognitive pathways
Source: Front Public Health. 2026 Sep 17;14:1933490. doi: 10.3389/fpubh.2026.1933490 (PMC13627094; doi:10.3389/fpubh.2026.1933490)
Supplement: Supplementary file 1 [file Table_1.DOCX]

**Supplementary Table S1. Spearman’s rank correlation coefficients among demographic, environmental, psychological, and activity-related variables**

| **Variable** | **1** | **2** | **3** | **4** | **5** | **6** | **7** | **8** | **9** | **10** | **11** | **12** | **13** |
| --- | --- | --- | --- | --- | --- | --- | --- | --- | --- | --- | --- | --- | --- |
| 1. Age | 1.000 | 0.108* | 0.001 | 0.055 | 0.051 | 0.038 | 0.022 | -0.010 | -0.074 | 0.049 | -0.023 | -0.055 | -0.001 |
| 2. ToP | 0.108* | 1.000 | -0.083 | 0.068 | 0.070 | -0.028 | 0.059 | -0.088 | -0.057 | -0.099 | 0.000 | 0.021 | -0.167** |
| 3. PfT | 0.001 | -0.083 | 1.000 | 0.032 | 0.038 | -0.143** | -0.121* | 0.037 | 0.015 | -0.017 | -0.038 | 0.047 | -0.035 |
| 4. Edu | 0.055 | 0.068 | 0.032 | 1.000 | 0.020 | 0.018 | -0.039 | -0.022 | -0.014 | -0.022 | 0.008 | 0.014 | 0.011 |
| 5. BMI | 0.051 | 0.070 | 0.038 | 0.020 | 1.000 | 0.024 | 0.071 | 0.040 | 0.044 | 0.006 | -0.011 | -0.028 | 0.083 |
| 6. ENV1 | 0.038 | -0.028 | -0.143** | 0.018 | 0.024 | 1.000 | 0.265*** | 0.353*** | 0.242*** | 0.314*** | -0.218*** | -0.135* | 0.411*** |
| 7. ENV2 | 0.022 | 0.059 | -0.121* | -0.039 | 0.071 | 0.265*** | 1.000 | 0.299*** | 0.318*** | 0.254*** | -0.296*** | -0.176*** | 0.288*** |
| 8. ENV3 | -0.010 | -0.088 | 0.037 | -0.022 | 0.040 | 0.353*** | 0.299*** | 1.000 | 0.271*** | 0.279*** | -0.217*** | -0.115* | 0.297*** |
| 9. AFF | -0.074 | -0.057 | 0.015 | -0.014 | 0.044 | 0.242*** | 0.318*** | 0.271*** | 1.000 | 0.281*** | -0.293*** | -0.141** | 0.309*** |
| 10. ENJ | 0.049 | -0.099 | -0.017 | -0.022 | 0.006 | 0.314*** | 0.254*** | 0.279*** | 0.281*** | 1.000 | -0.329*** | -0.129* | 0.224*** |
| 11. RNT | -0.023 | 0.000 | -0.038 | 0.008 | -0.011 | -0.218*** | -0.296*** | -0.217*** | -0.293*** | -0.329*** | 1.000 | 0.192*** | -0.316*** |
| 12. OUT | -0.055 | 0.021 | 0.047 | 0.014 | -0.028 | -0.135* | -0.176*** | -0.115* | -0.141** | -0.129* | 0.192*** | 1.000 | -0.170** |
| 13. TEA | -0.001 | -0.167** | -0.035 | 0.011 | 0.083 | 0.411*** | 0.288*** | 0.297*** | 0.309*** | 0.224*** | -0.316*** | -0.170** | 1.000 |

Note. Values are Spearman’s ρ coefficients. *p < 0.05, **p < 0.01, and ***p < 0.001.

1, Age; 2, trimester of pregnancy; 3, first-time pregnancy status; 4, educational level; 5, body mass index; 6, physical and sensory environment; 7, spatial and privacy environment; 8, social and organizational support; 9, positive affective state; 10, exercise enjoyment; 11, repetitive negative thinking; 12, psychological distress; 13, weekly Thermic Effect of Activity.
