# Supplementary material for: Associations between the prenatal exercise environment, exercise experience, and adherence: a cross-sectional study of affective and cognitive pathways
Source: Front Public Health. 2026 Sep 17;14:1933490. doi: 10.3389/fpubh.2026.1933490 (PMC13627094; doi:10.3389/fpubh.2026.1933490)
Supplement: Supplementary file 2 [file Table_2.DOCX]

**Supplementary Table S2.** Construct-level reliability, convergent validity, and discriminant validity of the first-order PLS-SEM measurement model

**Panel A. Construct-level reliability and convergent validity**

| **Construct** | **Retained items** | **Indicator loading range** | **Cronbach’s α** | **Composite reliability (rho_C)** | **AVE** |
| --- | --- | --- | --- | --- | --- |
| ENV1 | 11 | 0.768–0.813 | 0.942 | 0.950 | 0.632 |
| ENV2 | 3 | 0.849–0.861 | 0.817 | 0.891 | 0.732 |
| ENV3 | 3 | 0.851–0.862 | 0.818 | 0.892 | 0.733 |
| AFF | 6 | 0.763–0.817 | 0.886 | 0.913 | 0.635 |
| ENJ | 3 | 0.840–0.867 | 0.812 | 0.888 | 0.726 |
| RNT | 5 | 0.802–0.845 | 0.878 | 0.911 | 0.672 |
| OUT | 3 | 0.840–0.859 | 0.809 | 0.886 | 0.722 |

**Panel B. Heterotrait–monotrait ratio (HTMT) matrix**

| **Construct** | **AFF** | **ENJ** | **ENV1** | **ENV2** | **ENV3** | **OUT** | **RNT** |
| --- | --- | --- | --- | --- | --- | --- | --- |
| AFF | — |  |  |  |  |  |  |
| ENJ | 0.338 | — |  |  |  |  |  |
| ENV1 | 0.273 | 0.379 | — |  |  |  |  |
| ENV2 | 0.387 | 0.325 | 0.319 | — |  |  |  |
| ENV3 | 0.325 | 0.353 | 0.405 | 0.378 | — |  |  |
| OUT | 0.282 | 0.305 | 0.338 | 0.349 | 0.324 | — |  |
| RNT | 0.338 | 0.398 | 0.256 | 0.365 | 0.271 | 0.309 | — |

**Note.** ENV1, physical and sensory environment; ENV2, spatial and privacy environment; ENV3, social and organizational support; AFF, positive affective state; ENJ, exercise enjoyment; RNT, repetitive negative thinking; OUT, psychological distress; AVE, average variance extracted; HTMT, heterotrait–monotrait ratio. All HTMT values among the seven first-order constructs were below 0.85. The higher-order environmental construct (ENV), formed by ENV1, ENV2, and ENV3, was evaluated separately and is therefore not included as an independent first-order construct in this table.
