# Supplementary material for: Associations between the prenatal exercise environment, exercise experience, and adherence: a cross-sectional study of affective and cognitive pathways
Source: Front Public Health. 2026 Sep 17;14:1933490. doi: 10.3389/fpubh.2026.1933490 (PMC13627094; doi:10.3389/fpubh.2026.1933490)
Supplement: Supplementary file 3 [file Table_3.DOCX]

**Supplementary Table S3.** Complete item content, provenance, response format, and scoring of the Prenatal Exercise Environment and Experience Questionnaire (PEEQ)

| **Construct / subscale** | **Item code** | **Full item wording (Chinese / English)** | **Provenance and source / conceptual basis** |
| --- | --- | --- | --- |
| ENV1 Physical & Sensory Environment | S1 | 音乐节奏让我容易进入运动状态。 The music rhythm helped me get into the exercise flow. | Newly developed for this study; based on the PEED framework and relevant prenatal-exercise / environmental-psychology literature. No direct source instrument. |
|  | S2 | 音量让我感到舒适不过响。 The volume was comfortable, not too loud. |  |
|  | S3 | 我熟悉并喜欢这类音乐。 I was familiar with and liked this type of music. |  |
|  | L1 | 光线足够明亮但不刺眼。 Lighting was bright enough but not glaring. |  |
|  | L2 | 灯光色调让我放松。 The light color tone made me feel relaxed. |  |
|  | T1 | 温度让我感到舒适。 The temperature was comfortable. |  |
|  | T2 | 湿度适宜，没有黏腻或干燥感。 Humidity was appropriate, not sticky or dry. |  |
|  | Ar1 | 空气流通良好。 Air was well-ventilated. |  |
|  | Ar2 | 没有让我不适的气味。 There was no unpleasant smell. |  |
|  | N1 | 场地有植物、自然图片或窗外景观。 The place had natural elements (plants, nature pictures, outdoor view). |  |
|  | N2 | 这些自然元素让我更容易专注或放松。 These natural elements helped me focus or relax. |  |
| ENV2 Spatial & Privacy Environment | SP1 | 场地不拥挤，有足够空间活动。 The place was not crowded and had enough space to move. |  |
|  | SP2 | 我在场地中感到安全和私密。 I felt safe and had enough privacy. |  |
|  | SP3 | 更衣、饮水、休息都方便。 Facilities for changing, drinking, and resting were convenient. |  |
| ENV3 Social & Organizational Support | SS1 | 教练关注我的状态并调整训练。 The instructor paid attention to my condition and adjusted the exercise. |  |
|  | SS2 | 同伴或家人鼓励我坚持运动。 My peers or family encouraged me to continue. |  |
|  | SS3 | 运动形式（线上/线下）适合我的生活节奏。 The exercise format (online/offline) fit my daily schedule. |  |
| AFF Affect | A1 | 兴奋 Excited | Researcher-developed; informed by established positive- and negative-affect measurement approaches. No direct source instrument administered. |
|  | A2 | 愉快 Happy |  |
|  | A3 | 放松 Relaxed |  |
|  | A4* | 紧张 Nervous |  |
|  | A5* | 沮丧 Upset |  |
|  | A6* | 烦躁 Irritated |  |
| ENJ Exercise Enjoyment | E1 | 我享受这次运动。 I enjoyed this exercise. | Researcher-developed; informed by exercise-enjoyment literature. No direct source instrument administered. |
|  | E2 | 我愿意再做类似的运动。 I would like to do similar exercise again. |  |
|  | E3 | 我感到运动让我身心放松。 The exercise made me feel relaxed. |  |
| RNT Repetitive Negative Thinking | R1 | 我脑中反复出现担忧或负面想法。 I kept thinking about worries or negative thoughts. | Adapted and contextualized from the PTQ conceptual/item framework (Ehring et al., 2011) for thoughts occurring during prenatal exercise; complete PTQ not administered. |
|  | R2 | 我难以摆脱对身体感觉的担心。 I found it hard to stop worrying about body sensations. |  |
|  | R3 | 我被一些负面想法困扰。 Some negative thoughts kept bothering me. |  |
|  | R4 | 我的脑子里充满了重复的想法。 My mind was full of repetitive thoughts. |  |
|  | R5 | 我无法控制这些想法。 I couldn't control these thoughts. |  |
| OUT Psychological Distress | P1 | 我最近感到紧张或焦虑。 I have felt nervous or anxious recently. | Researcher-developed; informed by pregnancy-related anxiety concepts represented in the PRAQ (Van den Bergh, 1990); PRAQ not administered as a complete instrument. |
|  | P2* | 我最近能很好地控制压力。 I have been able to control stress well. | Researcher-developed; informed by the perceived-control-over-stress concept represented in the PSS (Cohen et al., 1983); PSS not administered as a complete instrument. |
|  | P3 | 我担心怀孕期间发生不好的事情。 I worry that something may go wrong during pregnancy. | Researcher-developed; informed by pregnancy-related anxiety concepts represented in the PRAQ (Van den Bergh, 1990); PRAQ not administered as a complete instrument. |

**Notes.**

All retained PEEQ items were assessed using 7-point Likert-type response scales. ENV1, ENV2, ENV3, ENJ, and OUT were rated from 1 (strongly disagree) to 7 (strongly agree); AFF was rated from 1 (very slightly) to 7 (very strongly); and RNT was rated from 1 (never) to 7 (very often). Items marked with an asterisk (*) were reverse-coded using 8 minus the original response. Construct scores were calculated as the mean of the corresponding retained items. Higher scores indicate more favorable environmental perceptions for ENV1–ENV3, a more positive affective state for AFF, greater exercise enjoyment for ENJ, more frequent repetitive negative thinking for RNT, and greater psychological distress for OUT. “Newly developed” denotes items generated for the present study; “informed by” denotes researcher-developed items based on concepts represented in prior literature or established measures without administration of the original instrument; and “adapted and contextualized from” denotes study-specific modification of an established conceptual/item framework for the prenatal-exercise context. Perceived exertion and the additional exercise-adherence/intention measures were administered separately and were not indicators of the final seven-factor PEEQ measurement model; therefore, they are not included in this table.
