# Supplementary material for: Associations between the prenatal exercise environment, exercise experience, and adherence: a cross-sectional study of affective and cognitive pathways
Source: Front Public Health. 2026 Sep 17;14:1933490. doi: 10.3389/fpubh.2026.1933490 (PMC13627094; doi:10.3389/fpubh.2026.1933490)
Supplement: Supplementary file 4 [file Table_4.DOCX]

**Supplementary Table S4. Complete Varimax-rotated factor-loading matrix for the initial 35 PEEQ candidate items**

| **Item** | **Factor 1 (ENV1)** | **Factor 2 (AFF)** | **Factor 3 (RNT)** | **Factor 4 (ENV2)** | **Factor 5 (OUT)** | **Factor 6 (ENV3)** | **Factor 7 (ENJ)** | **Communality** |
| --- | --- | --- | --- | --- | --- | --- | --- | --- |
| S1 | **0.764** | 0.089 | −0.036 | −0.007 | 0.115 | 0.161 | 0.039 | 0.634 |
| S2 | **0.747** | 0.018 | −0.023 | 0.081 | 0.080 | 0.069 | 0.138 | 0.596 |
| S3 | **0.767** | 0.072 | −0.105 | 0.128 | 0.098 | 0.039 | −0.014 | 0.632 |
| S4 (R) | −0.125 | −0.019 | −0.014 | −0.175 | −0.174 | 0.093 | −0.086 | 0.093 |
| L1 | **0.779** | 0.097 | −0.065 | 0.033 | 0.004 | 0.106 | 0.116 | 0.646 |
| L2 | **0.777** | 0.072 | −0.059 | −0.011 | 0.055 | 0.093 | 0.093 | 0.633 |
| T1 | **0.785** | 0.059 | −0.066 | 0.153 | 0.084 | 0.073 | 0.067 | 0.665 |
| T2 | **0.789** | 0.089 | −0.026 | 0.096 | 0.028 | 0.012 | 0.064 | 0.645 |
| Ar1 | **0.776** | 0.161 | −0.061 | 0.042 | 0.064 | 0.115 | 0.074 | 0.657 |
| Ar2 | **0.779** | 0.058 | −0.070 | 0.033 | 0.039 | 0.076 | 0.055 | 0.626 |
| N1 | **0.787** | 0.014 | −0.074 | 0.056 | 0.088 | 0.046 | 0.098 | 0.647 |
| N2 | **0.781** | 0.075 | −0.120 | 0.111 | 0.134 | 0.061 | 0.052 | 0.666 |
| SP1 | 0.126 | 0.204 | −0.101 | **0.788** | 0.136 | 0.129 | 0.041 | 0.725 |
| SP2 | 0.106 | 0.126 | −0.191 | **0.806** | 0.069 | 0.084 | 0.124 | 0.741 |
| SP3 | 0.150 | 0.118 | −0.112 | **0.805** | 0.065 | 0.126 | 0.029 | 0.717 |
| SS1 | 0.204 | 0.086 | −0.076 | 0.108 | 0.019 | **0.795** | 0.121 | 0.714 |
| SS2 | 0.202 | 0.159 | −0.088 | 0.043 | 0.125 | **0.812** | −0.003 | 0.750 |
| SS3 | 0.167 | 0.103 | −0.083 | 0.141 | 0.091 | **0.803** | 0.144 | 0.739 |
| A1 | 0.142 | **0.764** | −0.062 | 0.109 | 0.026 | 0.124 | 0.104 | 0.646 |
| A2 | 0.147 | **0.790** | −0.061 | 0.051 | 0.018 | 0.064 | 0.093 | 0.665 |
| A3 | 0.064 | **0.785** | −0.194 | 0.031 | 0.080 | 0.084 | −0.066 | 0.677 |
| A4 | −0.024 | **−0.763** | 0.065 | −0.190 | −0.097 | 0.038 | −0.072 | 0.638 |
| A5 | −0.215 | **−0.763** | 0.156 | 0.020 | −0.028 | −0.089 | −0.046 | 0.663 |
| A6 | 0.006 | **−0.771** | 0.066 | −0.144 | −0.104 | −0.073 | −0.162 | 0.662 |
| E1 | 0.159 | 0.113 | −0.155 | 0.127 | 0.056 | 0.032 | **0.807** | 0.733 |
| E2 | 0.197 | 0.135 | −0.173 | 0.068 | 0.142 | 0.075 | **0.769** | 0.709 |
| E3 | 0.157 | 0.112 | −0.124 | 0.035 | 0.041 | 0.154 | **0.813** | 0.740 |
| P1 | −0.169 | −0.061 | 0.121 | −0.068 | **−0.842** | −0.098 | −0.015 | 0.771 |
| P2 (R) | 0.169 | 0.099 | −0.079 | 0.087 | **0.788** | 0.128 | 0.087 | 0.697 |
| P3 | −0.088 | −0.123 | 0.122 | −0.100 | **−0.802** | −0.042 | −0.082 | 0.699 |
| R1 | −0.063 | −0.077 | **0.799** | −0.040 | −0.048 | −0.049 | −0.105 | 0.666 |
| R2 | −0.163 | −0.042 | **0.771** | −0.072 | −0.107 | −0.099 | −0.089 | 0.658 |
| R3 | −0.031 | −0.135 | **0.805** | −0.056 | −0.018 | −0.014 | −0.125 | 0.687 |
| R4 | −0.103 | −0.112 | **0.810** | −0.159 | −0.123 | −0.033 | −0.051 | 0.724 |
| R5 | −0.105 | −0.189 | **0.774** | −0.082 | −0.051 | −0.073 | −0.082 | 0.668 |

Note. Values represent the Varimax-rotated factor loadings from the initial EFA of 35 candidate items. The EFA was conducted using the original item responses before reverse coding; therefore, items subsequently reverse-coded for scoring may show factor loadings in the opposite direction. All loadings on the seven extracted factors are reported to display potential cross-loadings. Bold values indicate the largest absolute loading for each retained item. S4 showed a communality of 0.093 and no salient loading on any factor and was therefore removed before subsequent measurement-model evaluation.
