# Supplementary material for: Associations between the prenatal exercise environment, exercise experience, and adherence: a cross-sectional study of affective and cognitive pathways
Source: Front Public Health. 2026 Sep 17;14:1933490. doi: 10.3389/fpubh.2026.1933490 (PMC13627094; doi:10.3389/fpubh.2026.1933490)
Supplement: Supplementary file 5 [file Table_5.DOCX]

**Supplementary Table S5. Complete exercise-behavior, adherence, and intention measures**

| **No.** | **Exact administered wording** | **Response options** | **Coding/scoring and analytical use** |
| --- | --- | --- | --- |
| 1 | Types of Sports last 4 weeks | 1. Almost no exercise/bed rest; 2. Walking, daily housework, prenatal stretching; 3. Slow walking, prenatal yoga; 4. Brisk walking, prenatal aerobics, prenatal Pilates; 5. Stationary cycling, moderate aerobic exercise; 6. Swimming, jogging, fast cycling; 7. Ball games, dancing, strength training; 8. HIIT, running, vigorous exercise. | Recorded as one of eight predefined exercise-type categories (1–8). Exercise type was used together with reported exercise intensity to support matching with the corresponding activity in the 2024 Adult Compendium for estimation of weekly TEA. |
| 2 | How many times per week have you exercised on average in the past 4 weeks? | Open numerical response (times/week). | Recorded as weekly exercise frequency. Used directly in the weekly TEA calculation. |
| 3 | Average duration per session (minutes): | Open numerical response (minutes/session). | Recorded in minutes/session and converted to hours for the weekly TEA calculation. |
| 4 | Did you stop exercising for a week or more during the past 4 weeks? | No / Yes. | Recorded as an individual binary categorical measure of exercise interruption. No composite adherence score was calculated. This item was not included in the SEM or machine-learning analyses. |
| 5 | Do you plan to continue exercising in the next two weeks? | Yes / No. | Recorded as an individual binary categorical measure of short-term continuation plans. No composite intention score was calculated. This item was not included in the SEM or machine-learning analyses. |
| 6 | Please rate your intention to continue exercising (0–10). | 0–10; 0 = Not at all; 10 = Definitely will continue. | Recorded as a single-item 0–10 intention rating. No composite intention score was calculated. This item was not included in the SEM or machine-learning analyses. |

Note. These researcher-developed exercise-behavior/adherence/intention measures were administered separately from the final PEEQ measurement model and were not combined into psychometric constructs or composite adherence/intention scores. Exercise type, weekly exercise frequency, and session duration were used to support estimation of weekly TEA. The exercise-interruption and intention-related items were retained as individual descriptive measures and were not included in the subsequent SEM or machine-learning analyses.
